# Author Correction: Highly secreted tryptophanyl tRNA synthetase 1 as a potential theranostic target for hypercytokinemic severe sepsis

**DOI:** 10.1038/s44321-024-00030-4

**Published:** 2024-02-05

**Authors:** Yoon Tae Kim, Jin Won Huh, Yun Hui Choi, Hee Kyeong Yoon, Tram TT Nguyen, Eunho Chun, Geunyeol Jeong, Sunyoung Park, Sungwoo Ahn, Won-Kyu Lee, Young-Woock Noh, Kyoung Sun Lee, Hee-Sung Ahn, Cheolju Lee, Sang Min Lee, Kyung Su Kim, Gil Joon Suh, Kyeongman Jeon, Sunghoon Kim, Mirim Jin

**Affiliations:** 1 Department of Health Sciences and Technology, GAIHST, Gachon University, Incheon, Republic of Korea; 2 Department of Pulmonary and Critical Care Medicine, Asan Medical Center, University of Ulsan College of Medicine, Seoul, Republic of Korea; 3 R&D Center, MirimGENE, Incheon, Republic of Korea; 4 Lee Gil Ya Cancer and Diabetes Institute, Gachon University, Incheon, Republic of Korea; 5 Arnie Charbonneau Cancer Institute, University of Calgary, Calgary, AB Canada; 6 New Drug Development Center, Osong Medical Innovation Foundation, Cheongju, Republic of Korea; 7 Non-Clinical Evaluation Center, Osong Medical Innovation Foundation, Cheongju, Republic of Korea; 8 Convergence Medicine Research Center, Asan Institute for Life Sciences, Asan Medical Center, Seoul, Republic of Korea; 9 Chemical & Biological Integrative Research Center, Korea Institute of Science and Technology, Seoul, Republic of Korea; 10 Department of Internal Medicine, Gil Medical Center, College of Medicine, Gachon University, Incheon, Republic of Korea; 11 Department of Emergency Medicine, Seoul National University Hospital, Seoul, Republic of Korea; 12 Department of Emergency Medicine, Seoul National University College of Medicine, Seoul, Republic of Korea; 13 Division of Pulmonary and Critical Care Medicine, Department of Medicine, Samsung Medical Center, Sungkyunkwan University School of Medicine, Seoul, Republic of Korea; 14 Medicinal Bioconvergence Research Center, Institute for Artificial Intelligence and Biomedical Research, The interdisciplinary graduate program in integrative biotechnology, College of Pharmacy & College of Medicine, Gangnam Severance Hospital, Yonsei University, Incheon, Republic of Korea; 15 Department of Microbiology, College of Medicine, Gachon University, Incheon, Republic of Korea

## Abstract

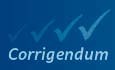

**Correction to:** *EMBO Molecular Medicine* (2023) 16:40–63. 10.1038/s44321-023-00004-y | Published online 14 December 2023

**There is an error in the Acknowledgements section of this article relating to a grant number for the Korea Health Technology R&D Project by the Korea Health Industry Development Institute (KHIDI) of the Ministry of Health & Welfare, Republic of Korea**.

The Acknowledgements section is corrected from:

This research was supported by the Bio & Medical Technology Development Program of the National Research Foundation (NRF) of the Korean government (MSIT), Republic of Korea, grant numbers NRF-2019M3E5D5064771, the Korea Health Technology R&D Project by the Korea Health Industry Development Institute (KHIDI) of the Ministry of Health & Welfare, Republic of Korea, grant number HI20C0015, and HI122C10883, Daegu-Gyeongbuk/Osong Medical Cluster R&D Project funded by the Ministry of Science and ICT, the Ministry of Trade, Industry and Energy, the Ministry of Health & Welfare, Republic of Korea, grant number HI19C0763, and Korea Drug Development Fund funded by Ministry of Science and ICT, Ministry of Trade, Industry, and Energy, and Ministry of Health and Welfare, Republic of Korea, grant number RS-2022-00166575.

To (see changes in bold)

This research was supported by the Bio & Medical Technology Development Program of the National Research Foundation (NRF) of the Korean government (MSIT), Republic of Korea, grant numbers NRF-2019M3E5D5064771, the Korea Health Technology R&D Project by the Korea Health Industry Development Institute (KHIDI) of the Ministry of Health & Welfare, Republic of Korea, grant number HI20C0015, and **HI22C1883**, Daegu-Gyeongbuk/Osong Medical Cluster R&D Project funded by the Ministry of Science and ICT, the Ministry of Trade, Industry and Energy, the Ministry of Health & Welfare, Republic of Korea, grant number HI19C0763, and Korea Drug Development Fund funded by Ministry of Science and ICT, Ministry of Trade, Industry, and Energy, and Ministry of Health and Welfare, Republic of Korea, grant number RS-2022-00166575.

This change does not affect the text or interpretation of the article.

