# Supplementary material for: Highly secreted tryptophanyl tRNA synthetase 1 as a potential theranostic target for hypercytokinemic severe sepsis
Source: EMBO Mol Med. 2023 Dec 14;16(1):40–63. doi: 10.1038/s44321-023-00004-y (PMC10883277; doi:10.1038/s44321-023-00004-y)
Supplement: Supplementary file 1 — Appendix [file 44321_2023_4_MOESM1_ESM.pdf]

Appendix

Highly secreted tryptophanyl tRNA synthetase 1 as a potential theranostic target for hypercytokinemic severe sepsis

Yoon Tae Kim, Jin Won Huh, Yun Hui Choi, Hee Kyeong Yoon, Tram T. T. Nguyen, Eunho Chun, Geunyeol Jeong, Sunyoung Park, Sungwoo Ahn, Won Kyu Lee, Young-Woock Noh, Kyoung Sun Lee, Hee Sung Ahn, Cheolju Lee, Sang Min Lee, Kyung Su Kim, Gil Joon Suh, Kyeongman Jeon, Sunghoon Kim, and Mirim Jin*

*Corresponding to: mirimj@gachon.ac.kr

Table of contents

Appendix Figure S1……………………………………………………………………….2

Appendix Figure S2……………………………………………………………………….3

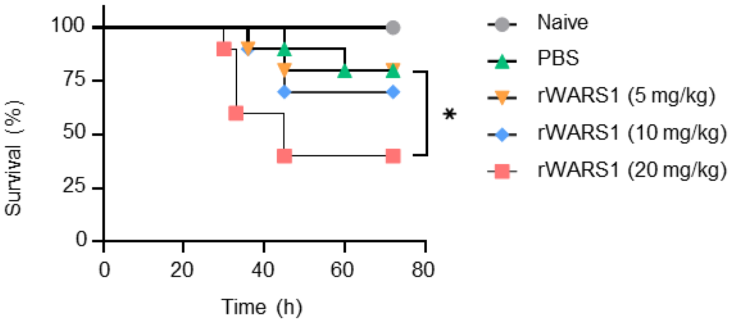

Appendix Fig S1. Analysis of survival rate following dose-dependent administration of recombinant WARS1 in mice with mild sepsis.

Kaplan-Meier survival plot for mice administered with PBS or recombinant WARS1 (5–20 mg/kg) after CS_{16mg} inoculation. Naïve mice were administered with PBS (n = 10). Statistical analysis is performed with log-rank test. *$p < 0.05$.

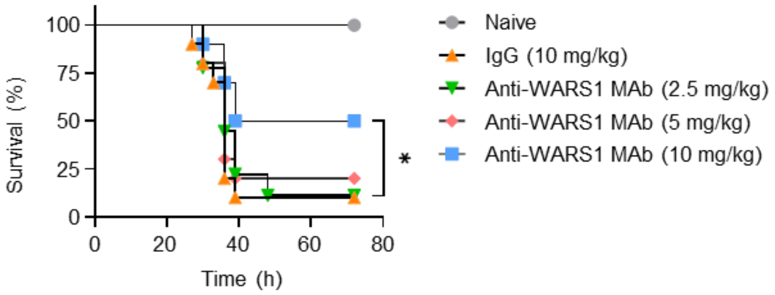

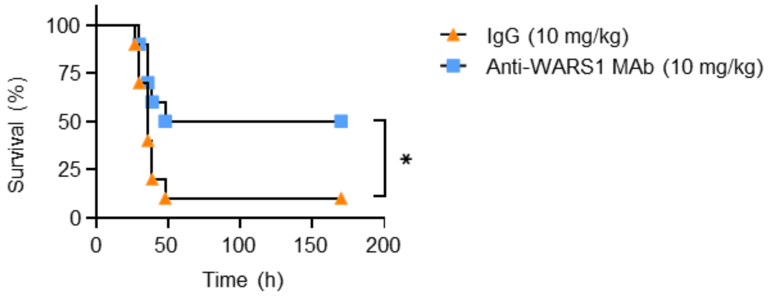

Appendix Figure S2. Analysis of survival rate following dose-dependent administration of anti-WARS1 MAb in mice with severe sepsis.

A. Kaplan-Meier survival plot for mice administered with IgG (10 mg/kg) or anti-WARS1 MAb (2.5–10 mg/kg) after CS_{20mg} inoculation. Naïve mice were administered with PBS (n = 10).

B. Kaplan-Meier survival plot up to 7 days for mice administered with IgG (10 mg/kg) or anti-WARS1 MAb (10 mg/kg) after CS_{20mg} inoculation (n = 10).

Data information: Statistical analysis is performed with log-rank test (A and B). *$p < 0.05$.
