# Supplementary material for: Highly secreted tryptophanyl tRNA synthetase 1 as a potential theranostic target for hypercytokinemic severe sepsis
Source: EMBO Mol Med. 2023 Dec 14;16(1):40–63. doi: 10.1038/s44321-023-00004-y (PMC10883277; doi:10.1038/s44321-023-00004-y)
Supplement: Supplementary file 3 — Table EV1 [file 44321_2023_4_MOESM3_ESM.docx]

| Characteristics | Sepsis (n=100) | Septic shock (n=89) | *p*-value |
| --- | --- | --- | --- |
| Age (years) | 62 (54-71) | 57 (47-68) | 0.060 |
| Sex (male), n (%) | 64 (64) | 63 (70) | 0.350 |
| SOFA score | 8 (6-12) | 16 (12-19) | <0.001 |
| Mortality, n (%) | 22 (22) | 58 (65.1) | <0.001 |
| **Comorbidities, n (%)** |  |  |  |
| Respirology | 10 (10) | 4 (4.5) | 0.170 |
| Cardiology | 34 (34) | 24 (27) | 0.340 |
| Gastrology | 9 (9) | 6 (6.7) | 0.600 |
| Hepatology | 16 (16) | 31 (34.8) | 0.004 |
| Endocrinology | 23 (23) | 23 (25.8) | 0.730 |
| Renal | 9 (9) | 8 (9.0) | >0.99 |
| Malignancy | 49 (49) | 57 (64) | 0.040 |
| Others | 20 (20) | 7 (7.8) | 0.020 |
| **Laboratory findings** |  |  |  |
| ANC | 9631 (5394-17445) | 7950 (1885-16187) | 0.070 |
| ANC count, n (%) |  |  |  |
| <500 | 10 (10) | 18 (20) |  |
| >500 | 90 (90) | 71 (80) |  |
| AMC | 584 (340-947) | 437 (128-796) | 0.01 |
| AMC count, n (%) |  |  |  |
| <500 | 42 (42) | 49 (55) |  |
| >500 | 58 (58) | 40 (45) |  |
| Lactate (mmol/L) | 1.6 (1.1-2.1) | 5 (3-9) | <0.001 |
| WARS1 (ng/mL) | 50.1 (23.3-102.4) | 82.6 (41.7-175.6) | <0.001 |
| PCT (ng/mL) | 1.1 (0.3-2.5) | 1.6 (0.6-5.3) | 0.002 |
| CRP (ng/mL) | 112.1 (70.6-155.6) | 110 (51.9-162.2) | 0.330 |
| IL-8/CXCL8 (pg/mL) | 32.3 (15.4-93.2) | 260.0 (96.8-1142) | <0.001 |
| CCL3/MIP-1α (pg/mL) | 9.9 (5.8-16.1) | 18.6 (8.9-32.7) | <0.001 |
| TNF-α (pg/mL) | 18.5 (11.1-30.8) | 28.0 (18.0-52.4) | <0.001 |
| IFN-γ (pg/mL) | 3.5 (1.6-7.9) | 4.6 (2.2-9.3) | 0.130 |
| **Site of infection, n (%)** |  |  | 0.690 |
| Pneumonia | 35 (35) | 40 (44.9) |  |
| Intraabdominal infection | 25 (25) | 24 (26.9) |  |
| Urinary tract infection | 12 (12) | 3 (3.3) |  |
| Others* | 28 (28) | 22 (24.7) |  |
| **Documented pathogens, n (%)** |  |  | 0.800 |
| Gram-negative | 37 (37) | 37 (41.6) |  |
| Gram-positive | 24 (24) | 22 (24.7) |  |
| Mixed | 10 (10) | 6 (6.7) |  |
| Anaerobes | 0 (0) | 3 (3.3) |  |
| Others | 1 (1) | 0 (0) |  |
| Unknown | 28 (28) | 21 (23.6) |  |
| **Bacteremia, n (%)** | 39 (39) | 43 (48.3) | 0.240 |

**Table EV1. Baseline characteristics of sepsis and septic shock in the sepsis cohort**

Data are presented as n (%) or median (interquartile range), unless indicated otherwise.

* Including CNS infection, soft tissue infection, catheter related infection, infected endocarditis, neutropenic fever, unknown. *p*-value of comparison between sepsis and septic shock patients

SOFA, Sequential Organ Failure Assessment; ANC, absolute neutrophil count; AMC, absolute monocyte count; WARS1, tryptophanyl-tRNA synthetase 1; PCT, procalcitonin; CRP, C-reactive protein; IL-8, interleukin 8; CXCL8, chemokine (C-X-C motif) ligand 8; CCL3, Chemokine (C-C motif) ligand 3; MIP-1α, macrophage inflammatory protein 1 alpha; TNF-α, tumor necrosis factor alpha; IFN-γ, interferon gamma.
