# Supplementary material for: Highly secreted tryptophanyl tRNA synthetase 1 as a potential theranostic target for hypercytokinemic severe sepsis
Source: EMBO Mol Med. 2023 Dec 14;16(1):40–63. doi: 10.1038/s44321-023-00004-y (PMC10883277; doi:10.1038/s44321-023-00004-y)
Supplement: Supplementary file 4 — Table EV2 [file 44321_2023_4_MOESM4_ESM.docx]

| Characteristics | Survivors (n=109) | Non-survivors (n=80) | *p*-value |
| --- | --- | --- | --- |
| Age (years) | 61 (47-69) | 60 (49-67) | 0.430 |
| Sex (male), n (%) | 74 (67.9) | 53 (66) | 0.880 |
| SOFA score | 9 (6.5-12.5) | 16 (12-19) | <0.001 |
| **Diagnosis, n (%)** |  |  |  |
| Sepsis | 78 (71.6) | 22 (27.5) |  |
| Septic shock | 31 (28.4) | 58 (72.5) |  |
| **Comorbidities, n (%)** |  |  |  |
| Respirology | 12 (11) | 2 (2.5) | 0.050 |
| Cardiology | 36 (33) | 22 (27.5) | 0.430 |
| Gastrology | 8 (7.3) | 7 (8.7) | 0.790 |
| Hepatology | 18 (16.5) | 29 (36.2) | 0.002 |
| Endocrinology | 29 (26.6) | 17 (21.2) | 0.490 |
| Renal | 13 (11.9) | 4 (5) | 0.130 |
| Malignancy | 49 (44.9) | 57 (71.2) | <0.001 |
| Others | 21 (19.3) | 6 (7.5) | 0.030 |
| **Laboratory findings** |  |  |  |
| ANC | 9300 (5313-17435) | 7411 (2159-16298) | 0.120 |
| ANC count, n (%) |  |  |  |
| <500 | 12 (11) | 16 (20) |  |
| >500 | 97 (88.9) | 64 (80) |  |
| AMC | 552 (291-880) | 437 (141-845) | 0.180 |
| AMC count, n (%) |  |  |  |
| <500 | 47 (43.1) | 44 (55) |  |
| >500 | 62 (56.9) | 36 (45) |  |
| Lactate (mmol/L) | 1.7 (1.2-2.8) | 5.3 (2.4-9.2) | <0.001 |
| WARS1 (ng/mL) | 53.3 (25.7-103.3) | 82.9 (36.3-259.0) | 0.007 |
| PCT (ng/mL) | 1.61 (0.39-3.41) | 1.26 (0.39-4.65) | 0.710 |
| CRP (ng/mL) | 113.2 (70.8-154.9) | 105.6 (50.3-163.3) | 0.240 |
| IL-8/CXCL8 (pg/mL) | 42.0 (16.8-164.8) | 269.1 (55.6-1251.0) | <0.001 |
| CCL3/MIP-1α (pg/mL) | 12.2 (6.2-24.8) | 12.4 (8.0-30.2) | 0.350 |
| TNF-α (pg/mL) | 22.0 (12.9-35.0) | 24.5 (15.4-46.3) | 0.120 |
| IFN-γ (pg/mL) | 4.6 (1.9-8.2) | 3.4 (1.8-9.3) | 0.640 |
| **Site of infection, n (%)** |  |  | 0.630 |
| Pneumonia | 33 (30.3) | 42 (52.5) |  |
| Intraabdominal infection | 32 (29.3) | 17 (21.2) |  |
| Urinary tract infection | 12 (11) | 3 (3.7) |  |
| Others* | 32 (29.3) | 18 (22.5) |  |
| **Documented pathogens, n (%)** |  |  | 0.790 |
| Gram-negative | 48 (44) | 26 (32.5) |  |
| Gram-positive | 20 (18.3) | 26 (32.5) |  |
| Mixed | 11 (10.1) | 5 (6.2) |  |
| Anaerobes | 1 (0.9) | 2 (2.5) |  |
| Others | 1 (0.9) | 0 (0) |  |
| Unknown | 28 (25.7) | 21 (26.2) |  |
| **Bacteremia, n (%)** | 48 (44) | 34 (42.5) | 0.880 |

**Table EV2. Baseline characteristics of survivors and non-survivors in the sepsis cohort**

Data are presented as n (%) or median (interquartile range), unless indicated otherwise.

* Including CNS infection, soft tissue infection, catheter related infection, infected endocarditis, neutropenic fever, unknown. *p*-value of comparison between sepsis and septic shock patients

SOFA, Sequential Organ Failure Assessment; ANC, absolute neutrophil count; AMC, absolute monocyte count; WARS1, tryptophanyl-tRNA synthetase 1; PCT, procalcitonin; CRP, C-reactive protein; IL-8, interleukin 8; CXCL8, chemokine (C-X-C motif) ligand 8; CCL3, Chemokine (C-C motif) ligand 3; MIP-1α, macrophage inflammatory protein 1 alpha; TNF-α, tumor necrosis factor alpha; IFN-γ, interferon gamma.
