# Supplementary material for: Highly secreted tryptophanyl tRNA synthetase 1 as a potential theranostic target for hypercytokinemic severe sepsis
Source: EMBO Mol Med. 2023 Dec 14;16(1):40–63. doi: 10.1038/s44321-023-00004-y (PMC10883277; doi:10.1038/s44321-023-00004-y)
Supplement: Supplementary file 5 — Table EV3 [file 44321_2023_4_MOESM5_ESM.docx]

|  | Odds ratio | 95% CI | *p*-value |
| --- | --- | --- | --- |
| Lactate | 1.333 | 1.167–1.523 | <0.001 |
| IL-8/CXCL8* | 1.006 | 1.001–1.010 | 0.023 |
| WARS1* | 1.026 | 1.001–1.052 | 0.039 |
| PCT | 0.896 | 0.797–1.007 | 0.065 |
| AMC | 1.000 | 0.999–1.001 | 0.070 |
| CCL3/MIP-1α | 0.990 | 0.977–1.003 | 0.149 |
| TNF-α | 1.002 | 0.999–1.005 | 0.210 |
| ANC | 1.000 | 0.999–1.000 | 0.274 |
| CRP* | 1.015 | 0.959–1.076 | 0.593 |
| IFN-γ | 1.003 | 0.985–1.022 | 0.714 |

**Table EV3. Multivariate analysis for predicting 28-day mortality in the sepsis cohort**

Data represent odds ratios and 95% confidence intervals (CI) by various parameters.

* ten-unit increment. *p*-values less than 0.05 were considered statistically significant.

IL-8, interleukin-8; CXCL8, chemokine (C-X-C motif) ligand 8; WARS1, tryptophanyl-tRNA synthetase 1; PCT, procalcitonin; AMC, absolute monocyte count; CCL3, Chemokine (C-C motif) ligand 3; MIP-1α, macrophage inflammatory protein 1 alpha; TNF-α, tumor necrosis factor alpha; ANC, absolute neutrophil count; CRP, C-reactive protein; IFN-γ, interferon gamma.
