# Supplementary material for: Highly secreted tryptophanyl tRNA synthetase 1 as a potential theranostic target for hypercytokinemic severe sepsis
Source: EMBO Mol Med. 2023 Dec 14;16(1):40–63. doi: 10.1038/s44321-023-00004-y (PMC10883277; doi:10.1038/s44321-023-00004-y)
Supplement: Supplementary file 6 — Table EV4 [file 44321_2023_4_MOESM6_ESM.docx]

| Species | Gene | Forward Sequence (5’-3’) | Reverse Sequence (3’-5’) |
| --- | --- | --- | --- |
| Human | IL-6 | CCGGGAACGAAAGAGAAGCT | GCGCTTGTGGAGAAGGAGTT |
|  | CXCL8/IL-8 | CTGGCCGTGGCTCTCTTG | CCTTGGCAAAACTGCACCTT |
|  | CCL3/MIP-1α | AGCCCACATTCCGTCACCTG | CGTGTCAGCAGCAAGTGATG |
|  | TNF-α | CCCAGGGACCTCTCTCTAATC | ATGGGCTACAGGCTTGTCACT |
|  | GAPDH | CACATGGCCTCCAAGGAGTAA | TGAGGGTCTCTCTCTTCCTCTTGT |
| Marmoset | IL-6 | CTGATCCAGTCCCTGCAGAA | CGAAGAGCCCTCAGACTGTT |
|  | CXCL8/IL-8 | AAGCTGGTTCTGGCTCTCTT | TTCTGTATTGGCGCAGTGTG |
|  | TNF-α | CTCCCAGGTCCTCTTCAAGG | ATGGCAGAGAGGAGGTTGAC |
|  | GAPDH | CCAGAACATCATCCCTGCCT | CCTGCTTCACCACCTTCTTG |
| Mouse | CCL2/MCP-1 | TGGAGCATCCACGTGTTGGC | ACTACAGCTTCTTTGGGACA |
|  | CCL7/MCP-3 | CCACATGCTGCTATGTCAAGA | ACACCGACTACTGGTGATCCT |
|  | CXCL1/KC | ACTGCACCCAAACCGAAGTC | TGGGGACACCTTTTAGCATCTT |
|  | CXCL5/ENA-78 | GTTCCATCTCGCCATTCATGC | GCGGCTATGACTGAGGAAGG |
|  | CXCL13/BLC | GGCCACGGTATTCTGGAAG | ACCGACAACAGTTGAAATCACTC |
|  | GAPDH | TGGCAAAGTGGAGATTGTTG | CATTCTCGGCCTTGACTGTG |

**Table EV4. Primers for quantitative real-time (qRT)-PCR**
