# Supplementary figures and images for: Highly secreted tryptophanyl tRNA synthetase 1 as a potential theranostic target for hypercytokinemic severe sepsis
Source: EMBO Mol Med. 2023 Dec 14;16(1):40–63. doi: 10.1038/s44321-023-00004-y (PMC10883277; doi:10.1038/s44321-023-00004-y)

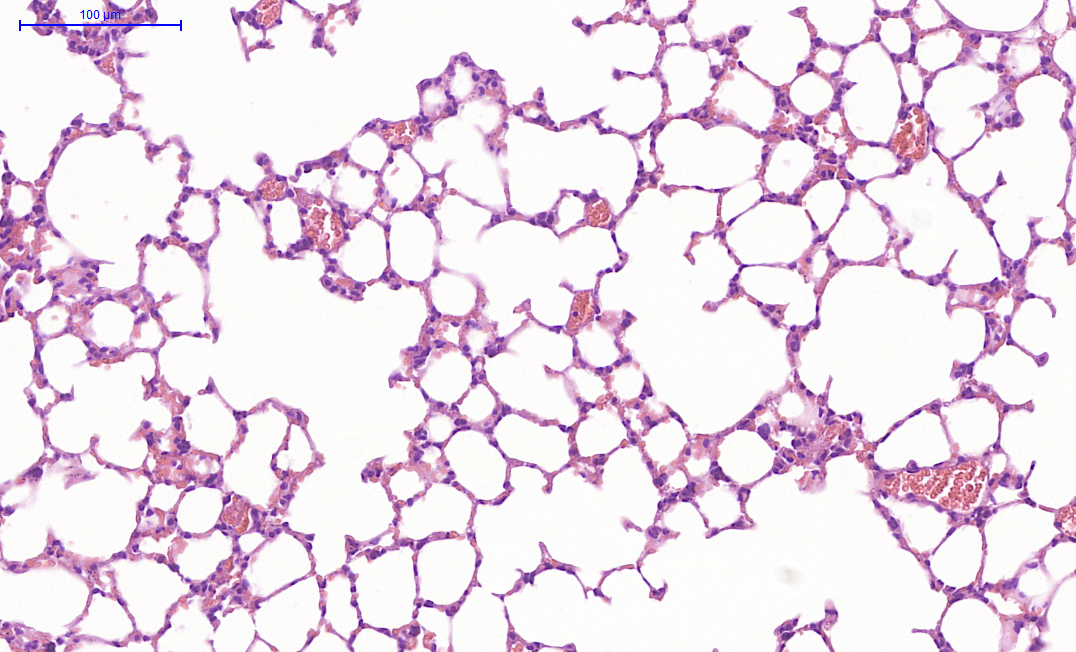

Supplement: Supplementary file 13 — Source Data Fig. 7 [file 44321_2023_4_MOESM13_ESM.zip › EMM-2023-18106_Figure_Source_Data_files_7/7A/Figure 7A - lung H&E (anti-WARS1 MAb).tiff]

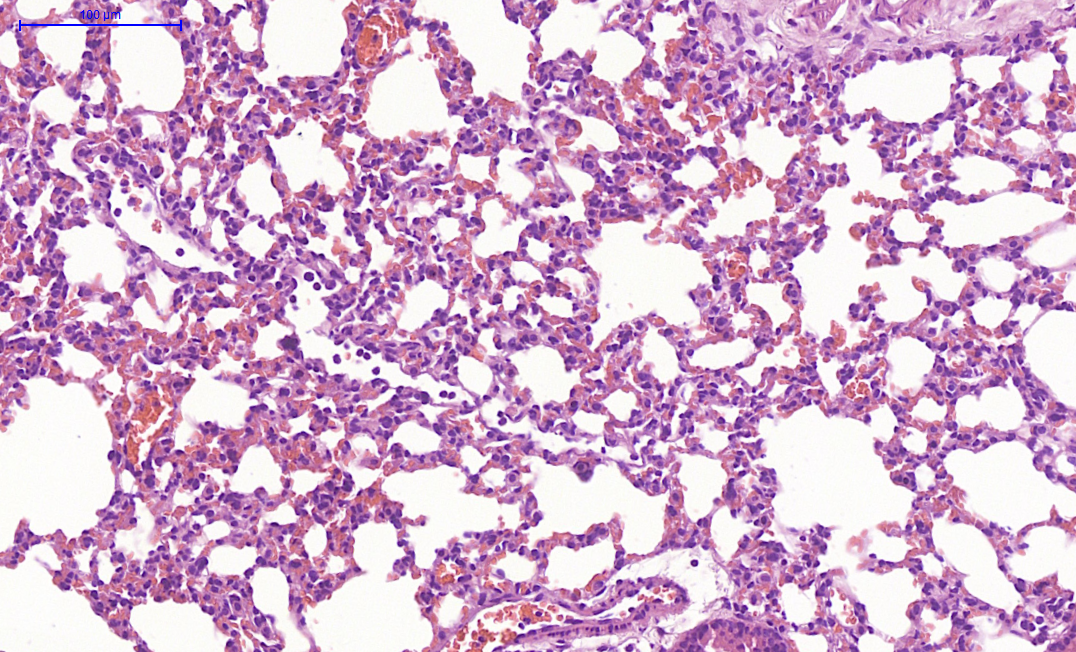

Supplement: Supplementary file 13 — Source Data Fig. 7 [file 44321_2023_4_MOESM13_ESM.zip › EMM-2023-18106_Figure_Source_Data_files_7/7A/Figure 7A - lung H&E (control IgG).tiff]

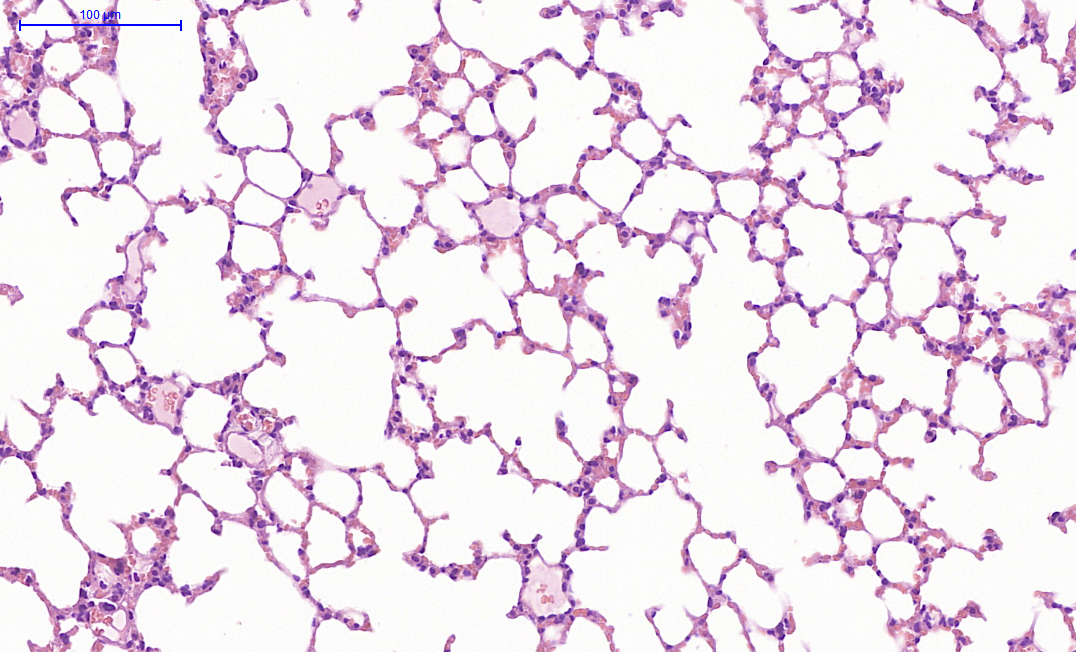

Supplement: Supplementary file 13 — Source Data Fig. 7 [file 44321_2023_4_MOESM13_ESM.zip › EMM-2023-18106_Figure_Source_Data_files_7/7A/Figure 7A - lung H&E (naive).tiff]

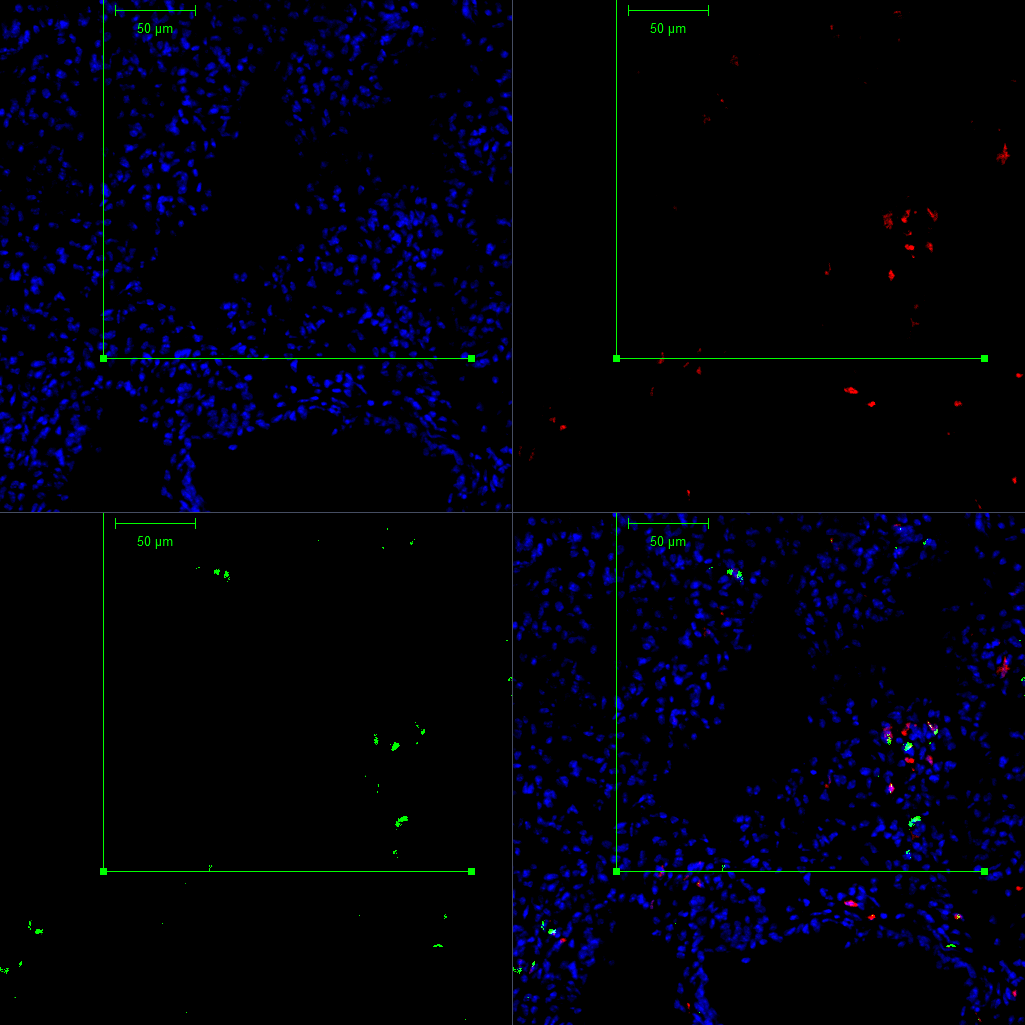

Supplement: Supplementary file 13 — Source Data Fig. 7 [file 44321_2023_4_MOESM13_ESM.zip › EMM-2023-18106_Figure_Source_Data_files_7/7B/Figure 7B - lung IF (anti-WARS1 MAb).tiff]

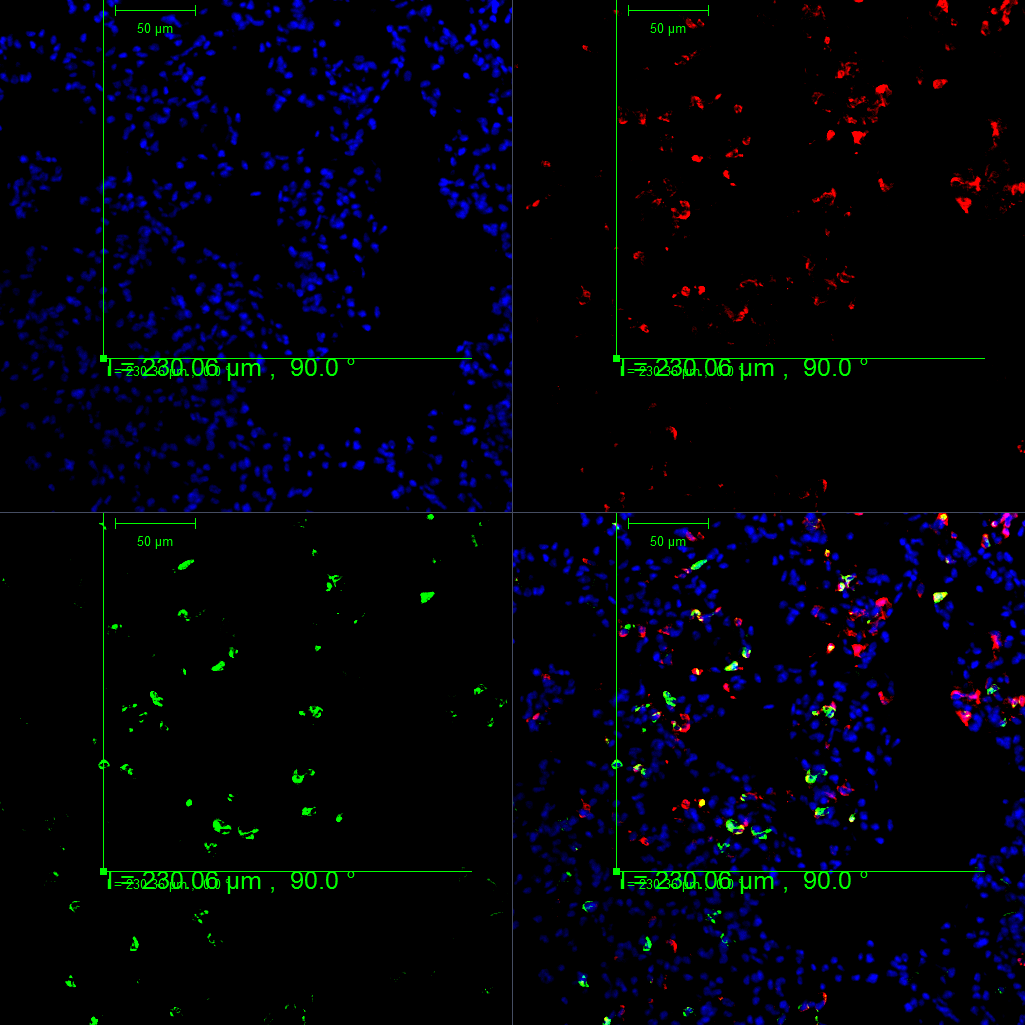

Supplement: Supplementary file 13 — Source Data Fig. 7 [file 44321_2023_4_MOESM13_ESM.zip › EMM-2023-18106_Figure_Source_Data_files_7/7B/Figure 7B - lung IF (control IgG).tiff]

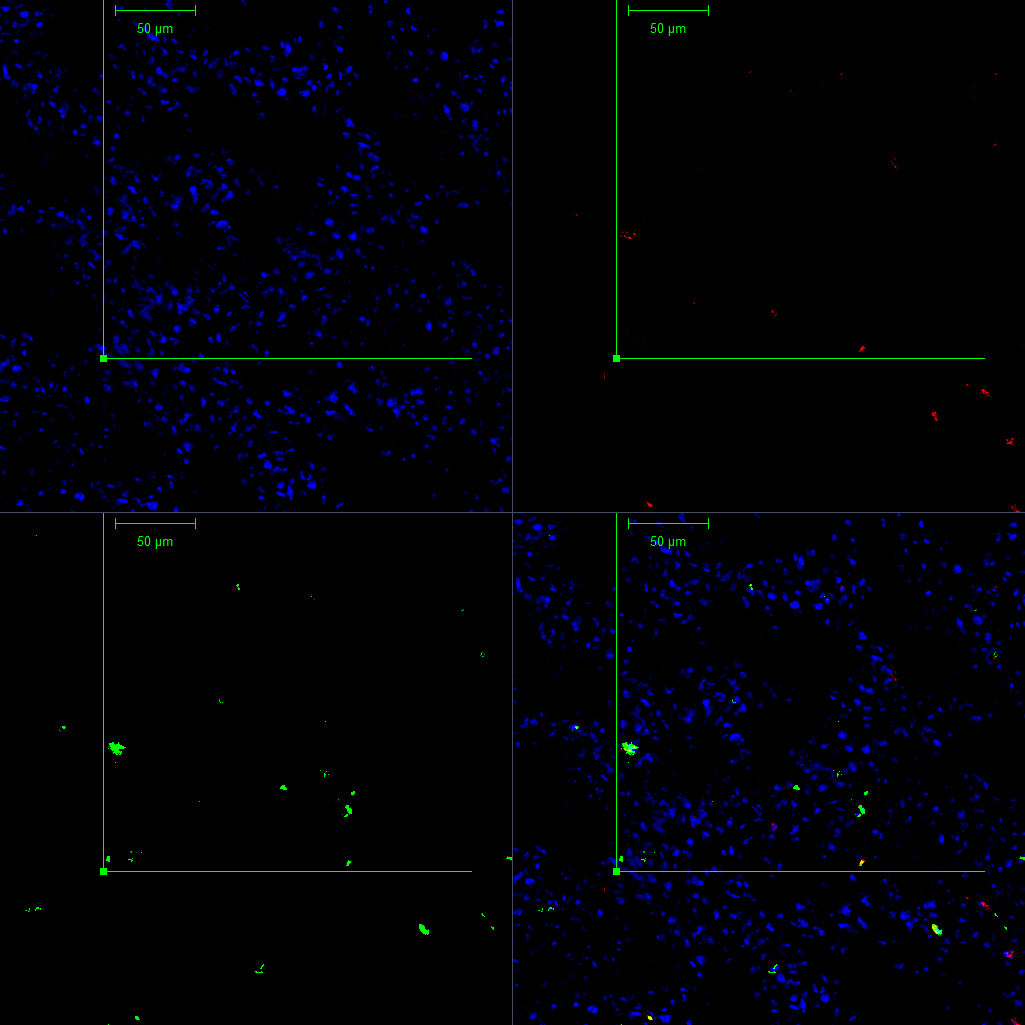

Supplement: Supplementary file 13 — Source Data Fig. 7 [file 44321_2023_4_MOESM13_ESM.zip › EMM-2023-18106_Figure_Source_Data_files_7/7B/Figure 7B - lung IF (naive).tiff]

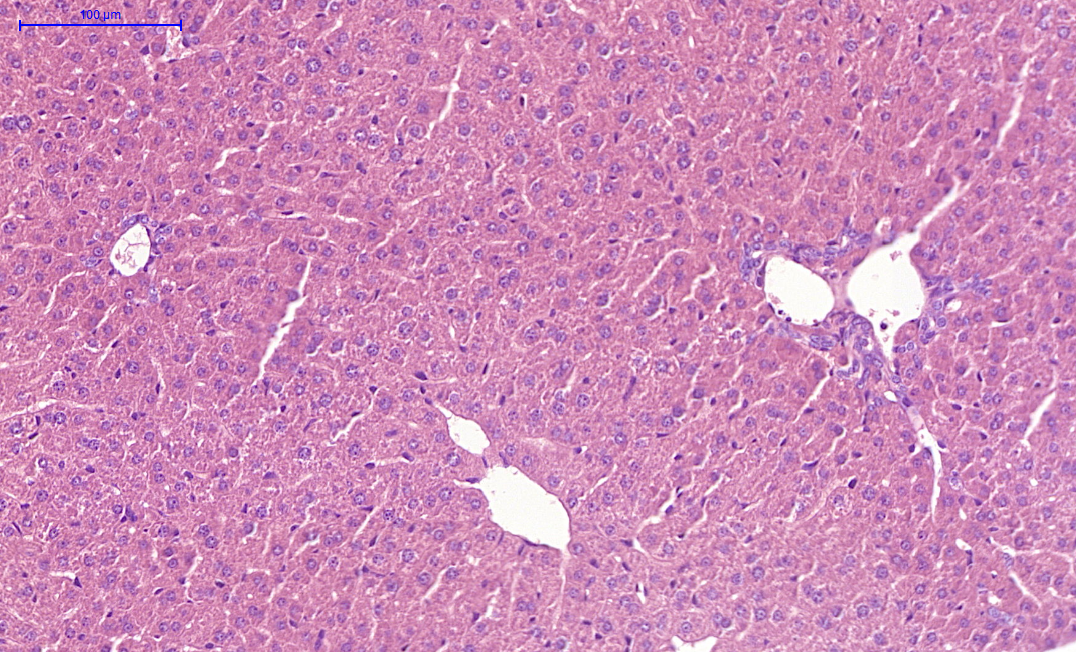

Supplement: Supplementary file 13 — Source Data Fig. 7 [file 44321_2023_4_MOESM13_ESM.zip › EMM-2023-18106_Figure_Source_Data_files_7/7E/Figure 7E - liver H&E (anti-WARS1 MAb).tiff]

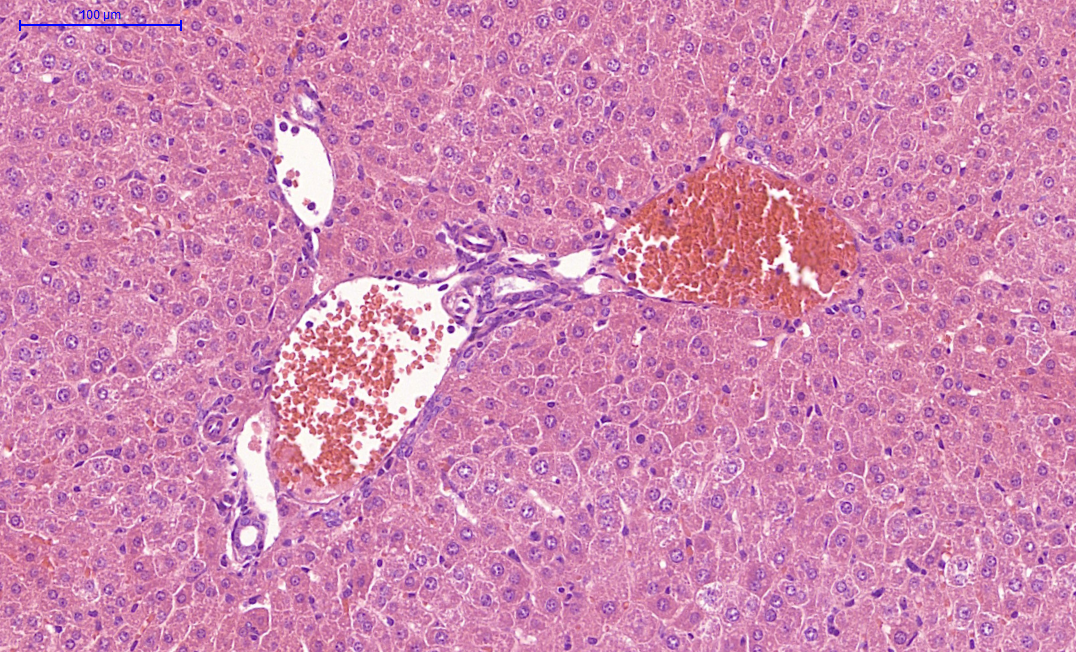

Supplement: Supplementary file 13 — Source Data Fig. 7 [file 44321_2023_4_MOESM13_ESM.zip › EMM-2023-18106_Figure_Source_Data_files_7/7E/Figure 7E - liver H&E (control IgG).tiff]

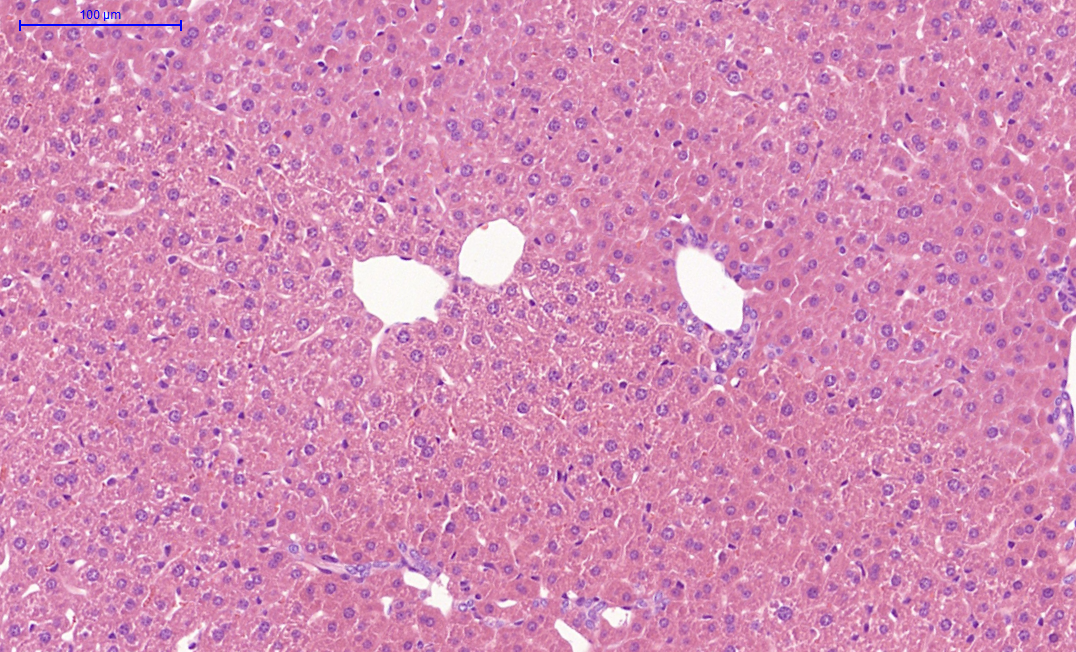

Supplement: Supplementary file 13 — Source Data Fig. 7 [file 44321_2023_4_MOESM13_ESM.zip › EMM-2023-18106_Figure_Source_Data_files_7/7E/Figure 7E - liver H&E (naive).tiff]

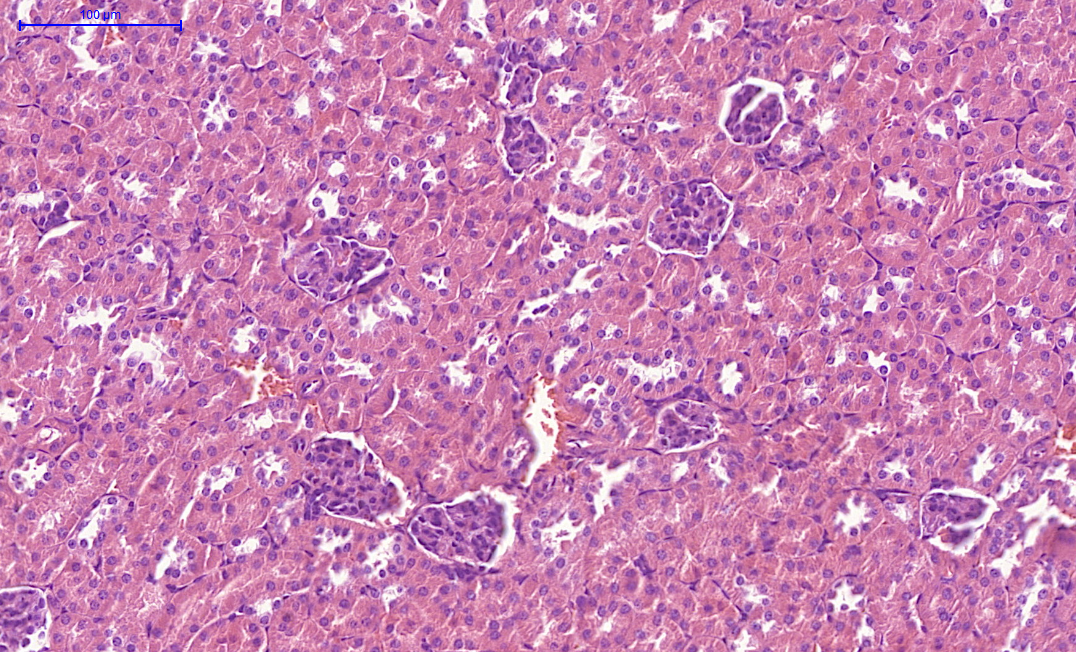

Supplement: Supplementary file 13 — Source Data Fig. 7 [file 44321_2023_4_MOESM13_ESM.zip › EMM-2023-18106_Figure_Source_Data_files_7/7F/Figure 7F - kideny H&E (anti-WARS1 MAb)1.tiff]

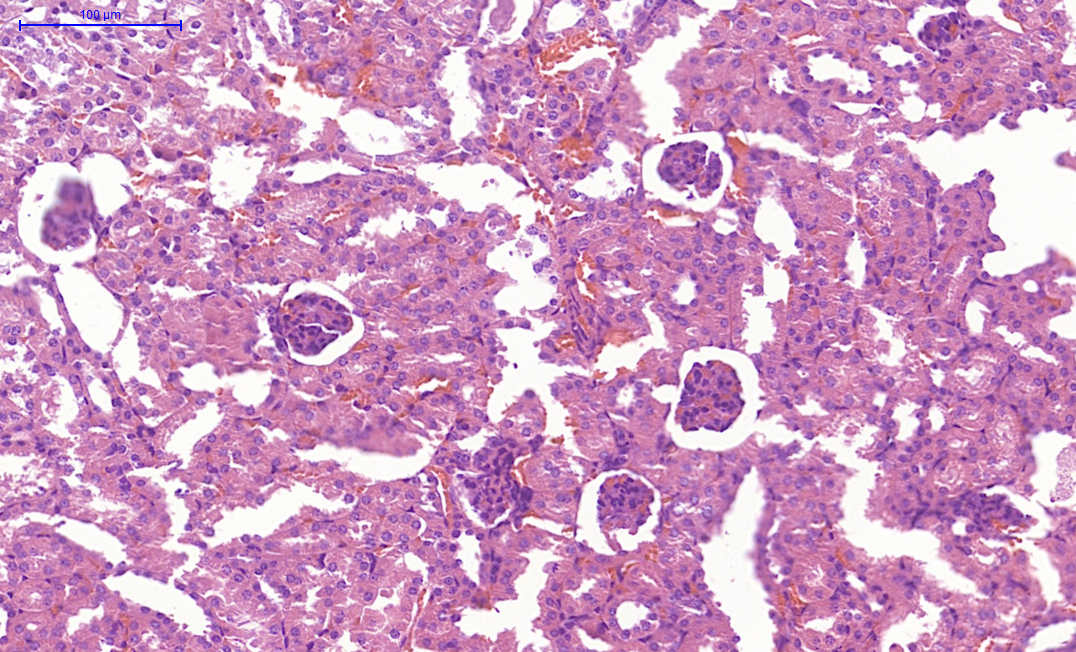

Supplement: Supplementary file 13 — Source Data Fig. 7 [file 44321_2023_4_MOESM13_ESM.zip › EMM-2023-18106_Figure_Source_Data_files_7/7F/Figure 7F - kidney H&E (control IgG).tiff]

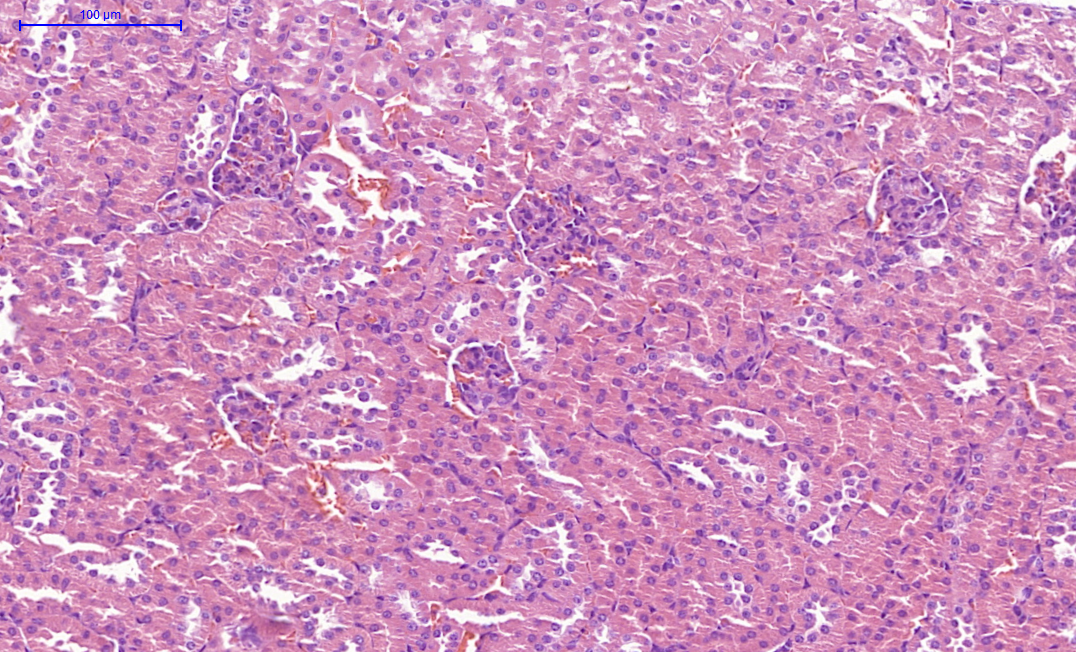

Supplement: Supplementary file 13 — Source Data Fig. 7 [file 44321_2023_4_MOESM13_ESM.zip › EMM-2023-18106_Figure_Source_Data_files_7/7F/Figure 7F - kidney H&E (naive).tiff]
