# Supplementary material for: Highly secreted tryptophanyl tRNA synthetase 1 as a potential theranostic target for hypercytokinemic severe sepsis
Source: EMBO Mol Med. 2023 Dec 14;16(1):40–63. doi: 10.1038/s44321-023-00004-y (PMC10883277; doi:10.1038/s44321-023-00004-y)
Supplement: Supplementary file 15 — Expanded View Figures [file 44321_2023_4_MOESM15_ESM.pdf]

Expanded View Figures

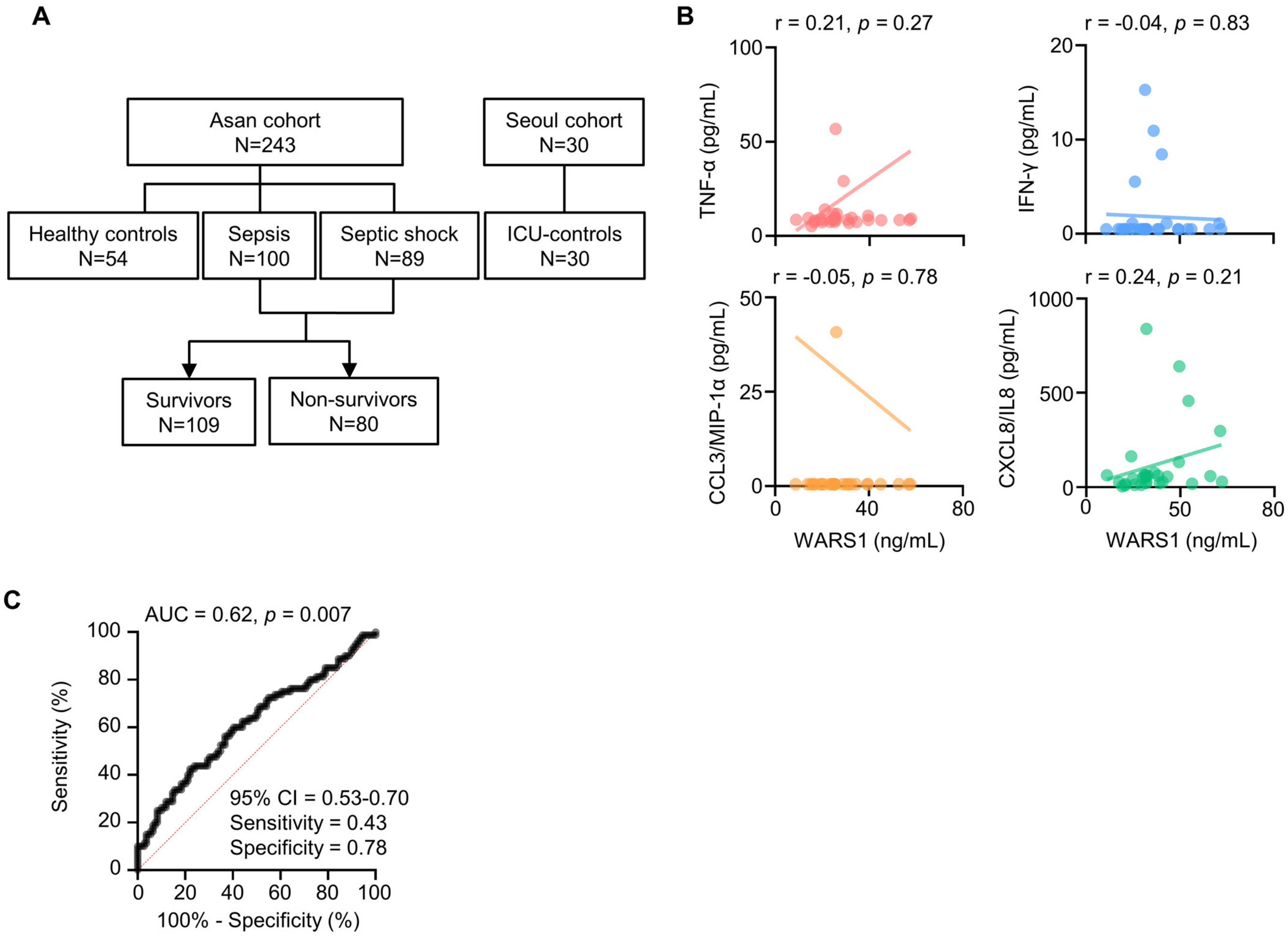

Figure EV1. Sepsis and ICU cohort study.

(**A**) Flowchart of the sepsis and ICU cohort population in Asan Medical Center and Seoul National University Hospital. (**B**) Correlation between WARS1 levels and cytokine and chemokine levels in the ICU controls ($n = 30$). Individual correlation results are reported with linear regression lines. (**C**) Receiver operating characteristic (ROC) analysis by cut-off value of WARS1 between survivors and non-survivors. Data information: Statistical analysis is performed with Pearson's correlation coefficient test (**B**), and ROC test (**C**).

© The Author(s)

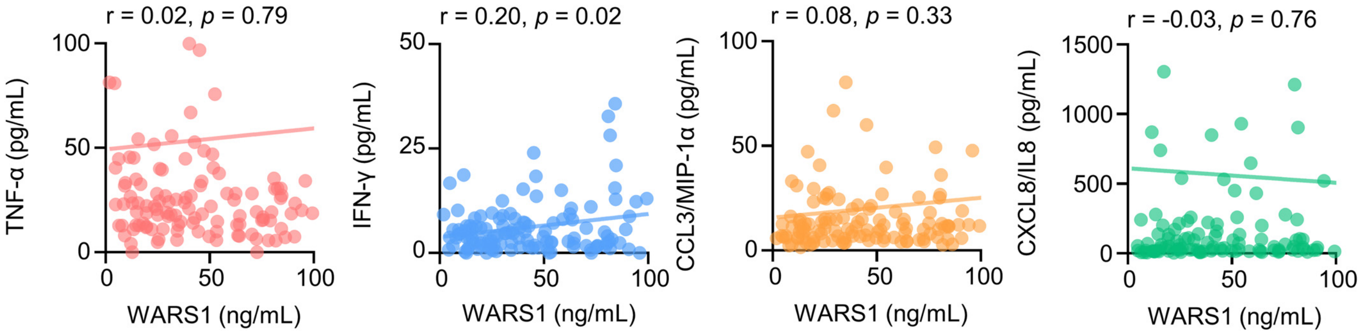

Figure EV2. The WARS1^{low} group showed no positive correlation with cytokine and chemokine levels.

Correlation between WARS1 levels and cytokine and chemokine levels in the WARS1^{low} ($n = 130$) group (stratified below 106.3 ng/mL). Individual correlation results are reported with linear regression lines. Statistical analysis is performed with Pearson's correlation coefficient test.

 © The Author(s)

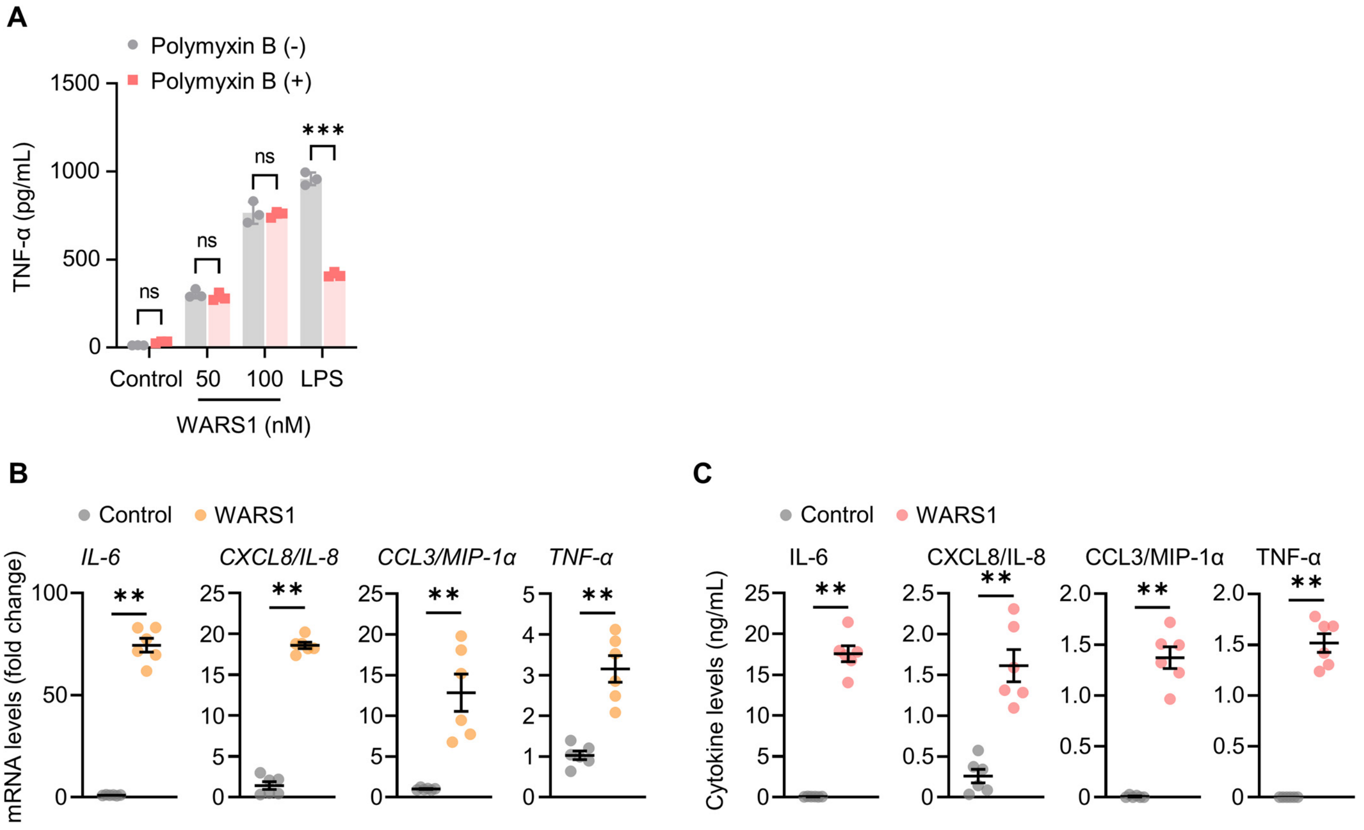

Figure EV3. Effect of WARS1-induced pro-inflammatory response in hPBMCs.

(**A**) TNF-α levels in the supernatant of PMA-differentiated THP-1 cells ($n = 3$) treated with hFL-WARS1 (50–100 nM) or LPS (100 ng/mL) with or without polymyxin B (50 μg/mL) for 9 h. (**B**) Gene expression of cytokine and chemokine in hPBMCs ($n = 6$) treated with hFL-WARS1 (50 nM) for 6 h. (**C**) Levels of cytokine and chemokine in the supernatants of hPBMCs ($n = 6$) treated with hFL-WARS1 (50 nM) for 6 h. Data information: Data are presented as mean ± SD (**A**), and ± SEM (**B**, **C**). Statistical analysis is performed with ANOVA with Bonferroni corrections (**A**), and Mann–Whitney *U*-test (**B**, **C**). ns, not significant; $^{**}p < 0.01$, $^{***}p < 0.001$.

© The Author(s)

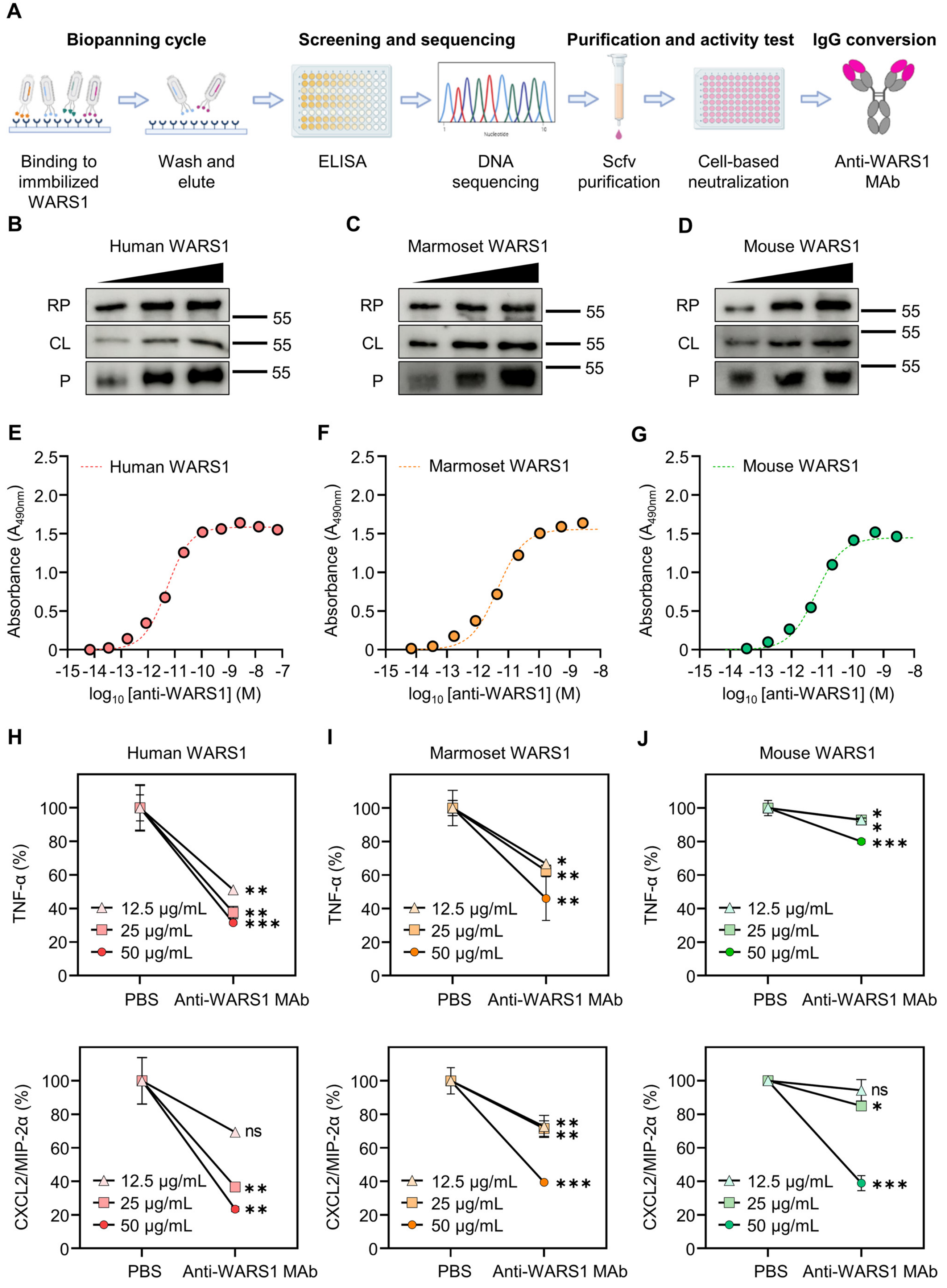
A
Biopanning cycle
Screening and sequencing
Purification and activity test
IgG conversion
Binding to immbilized WARS1
Wash and elute
ELISA
DNA sequencing
Scfv purification
Cell-based neutralization
Anti-WARS1 MAb
B
Human WARS1
C
Marmoset WARS1
D
Mouse WARS1
RP
CL
P
55
E
Human WARS1
F
Marmoset WARS1
G
Mouse WARS1
Absorbance (A_{490nm})
log10 [anti-WARS1] (M)
H
Human WARS1
I
Marmoset WARS1
J
Mouse WARS1
TNF-α (%)
12.5 μg/mL
25 μg/mL
50 μg/mL
PBS
Anti-WARS1 MAb
CXCL2/MIP-2α (%)
ns

 © The Author(s)

Figure EV4. Generation and neutralizing effect of anti-WARS1 MAb.

(**A**) Production process of WARS1 monoclonal antibody. After selectively isolating a clone that binds to WARS1 from the phage library (biopanning cycle), affinity and specificity were assessed, followed by DNA screening and sequencing. Then, the purified scfv was used for the neutralization test (purification and activity test) and finally converted to IgG (IgG conversion). (**B–D**) Immunoblot for human (**B**), marmoset (**C**), and mouse (**D**) WARS1 with anti-WARS1 MAb in recombinant full-length protein (RP), cell lysate (CL), and plasma (P). (**E–G**) Binding affinity of anti-WARS1 MAb to recombinant human (**E**), marmoset (**F**), and mouse (**G**) WARS1. (**H–J**) Levels of TNF-α and CXCL2/MIP-2α in the supernatant. J774A.1 cells ($n = 3$) were treated with a mixture of human (**H**), marmoset (**I**), and mouse (**J**) WARS1 and anti-WARS1 MAb or isotype IgG for 16 h. Data information: Data are presented as mean ± SD (**H–J**). Statistical analysis is performed with ANOVA with Bonferroni corrections (**H–J**). ns, not significant; *$p < 0.05$, **$p < 0.01$, ***$p < 0.001$.

© The Author(s)

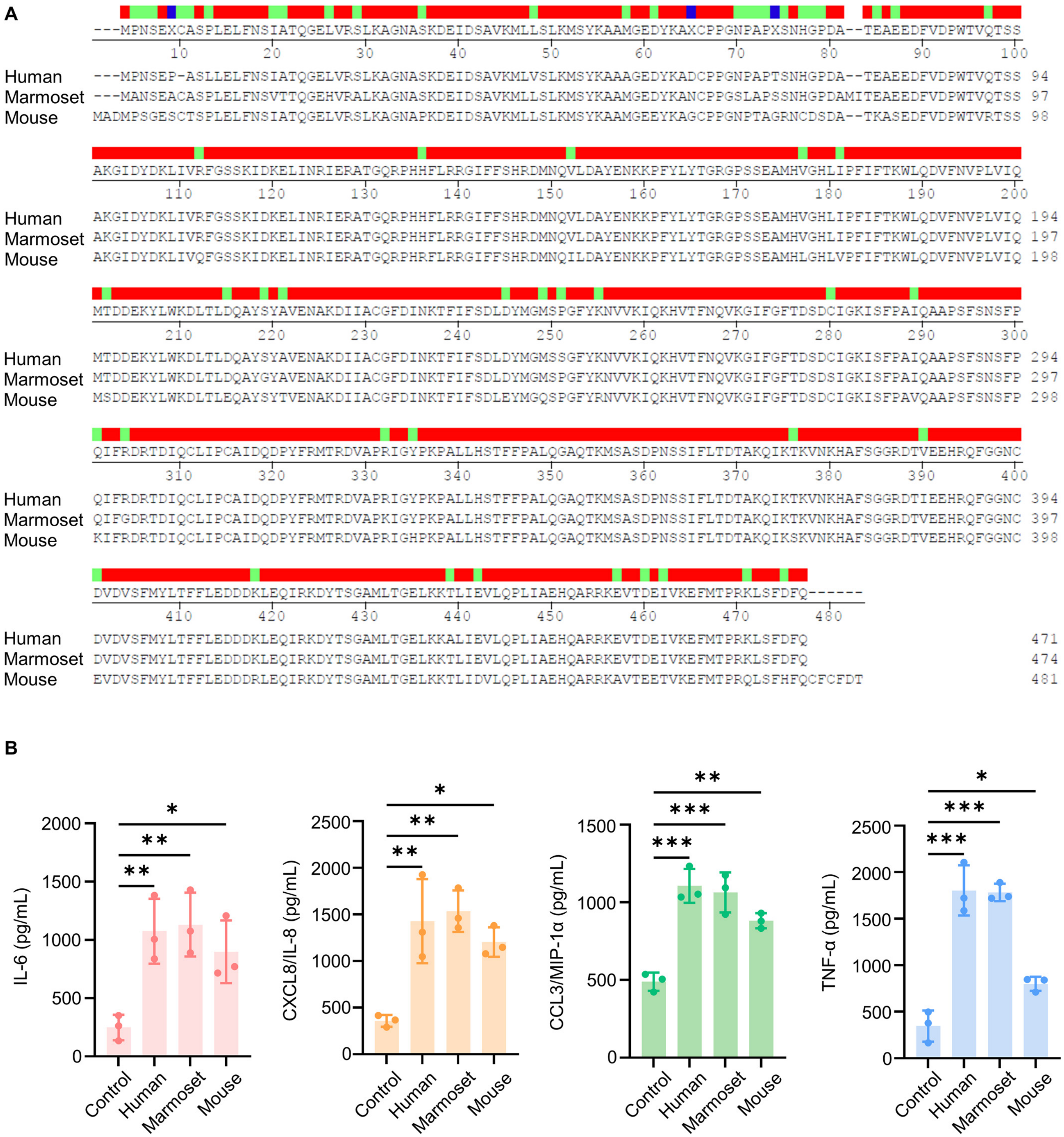

Figure EV5. Homology between human, marmoset, and mouse WARS1.

(**A**) Similarity of protein sequence between human, marmoset, and mouse FL-WARS1. (**B**) Levels of cytokine and chemokine in the supernatant of PMA-differentiated THP-1 cells ($n = 3$) treated with human, marmoset, and mouse WARS1 (50 nM) for 18 h. Data information: Data are presented as mean ± SD (**B**). Statistical analysis is performed with ANOVA with Bonferroni corrections (**B**). $^*p < 0.05$, $^{**}p < 0.01$, $^{***}p < 0.001$.

 © The Author(s)
